# Hepatocellular Carcinoma and Obesity, Type 2 Diabetes Mellitus, Cardiovascular Disease: Causing Factors, Molecular Links, and Treatment Options

**DOI:** 10.3389/fendo.2021.808526

**Published:** 2021-12-23

**Authors:** Chunye Zhang, Shuai Liu, Ming Yang

**Affiliations:** ^1^ Department of Veterinary Pathobiology, University of Missouri, Columbia, MO, United States; ^2^ The First Affiliated Hospital, Zhejiang University, Hangzhou, China; ^3^ Department of Surgery, University of Missouri, Columbia, MO, United States

**Keywords:** hepatocellular carcinoma, obesity, type 2 diabetes mellitus, cardiovascular diseases, nonalcoholic fatty liver disease, signaling pathway, treatment

## Abstract

Hepatocellular carcinoma (HCC) is the most common type of primary liver cancer, which will affect more than a million people by the year 2025. However, current treatment options have limited benefits. Nonalcoholic fatty liver disease (NAFLD) is the fastest growing factor that causes HCC in western countries, including the United States. In addition, NAFLD co-morbidities including obesity, type 2 diabetes mellitus (T2DM), and cardiovascular diseases (CVDs) promote HCC development. Alteration of metabolites and inflammation in the tumor microenvironment plays a pivotal role in HCC progression. However, the underlying molecular mechanisms are still not totally clear. Herein, in this review, we explored the latest molecules that are involved in obesity, T2DM, and CVDs-mediated progression of HCC, as they share some common pathologic features. Meanwhile, several therapeutic options by targeting these key factors and molecules were discussed for HCC treatment. Overall, obesity, T2DM, and CVDs as chronic metabolic disease factors are tightly implicated in the development of HCC and its progression. Molecules and factors involved in these NAFLD comorbidities are potential therapeutic targets for HCC treatment.

## Introduction

Hepatocellular carcinoma is the most common type of primary liver cancer, with a global case number larger than one million by 2025 (1). Factors including hepatitis viral infection, nonalcoholic fatty liver disease (NAFLD), alcohol abuse, and dietary toxins (e.g., aflatoxins) can cause the initiation and development of HCC (2). Both genetic and epigenetic factors can promote HCC progression (3), such as mutation of programmed cell death-1 (PDCD1, rs10204525 C > T mutation) and DNA methylation.

NAFLD is the fastest growing factor that causes HCC in western countries, including the United States (4). Many metabolic disorders, including obesity, type 2 diabetes mellitus (T2DM), and cardiovascular diseases (CVDs), are comorbidities of NAFLD or its advanced stage nonalcoholic steatohepatitis (NASH) (5). Gut microbiota and their-associated factors such as metabolites and components play important roles in the pathogenesis of obesity, T2DM, CVDs, NAFLD, and HCC (6–9). For example, feeding a high fat/high cholesterol diet (HFHC) can lead to fatty liver, NASH, fibrosis, and subsequential HCC in mice (10). Gut microbiota such as genera *Mucispirillum* and *Desulfovibrio* were increased, while genera *Bifidobacterium* and *Bacteroides* were dramatically decreased in HFHC-fed mice. In addition, gut microbial metabolites taurocholic acid and 3-indolepropionic acid were increased and decreased, separately, during NAFLD-HCC development (10). Overgrowth of nonvirulent lipopolysaccharide (LPS)-producing bacterial strains, such as *Enterobacter cloacae*, *Escherichia coli*, and *Klebsiella pneumoniae* from obese patients with severe fatty liver can induce NAFLD in germ-free mice fed a high-fat diet (HFD), but not HFD alone (11).

Chronic intestinal inflammation and malfunction of gut barrier associated with change of gut microbiota impact enteric hormones, adiposity, insulin resistance, and metabolic functions of intestine and other organs, such as *via* G protein-coupled receptors (12, 13). Accumulating evidence shows that GPCR signaling pathway play critically important roles in obesity, T2DM, and CVDs (14, 15). For example, phenylacetylglutamine, a gut-microbiota derived metabolite, can modulate cellular functions during CVD *via* GPCRs, such as α2A, α2B, and β2-adrenergic receptors (16). In addition to gut microbiota, several other factors such as chronic inflammation, insulin resistance, alteration of metabolites have been reported to be associated with obesity, T2DM, CVDs, as well as NAFLD, which can induce HCC initiation and progression. However, in specific situation, the risk factor may have different effect on these comorbidities. For example, genetic risk alleles (e.g., 148Met in the patatin-like phospholipase domain-containing protein 3, PNPLA3) in NAFLD are associated with protecting function in CVDs (17).

Herein, in this review, we summarize the relationships of obesity, T2DM, and CVDs with HCC, and uncover some signaling pathways and treatment options. The causing factors and treatment options of NAFLD-related HCC have been reported in another paper recently (3).

## Obesity and HCC

Obesity is a causing factor for many cancers (18), including HCC with a moderate magnitude in obesity-associated cancers. For example, dysbiosis of gut microbiota is shown to be associated with obesity, resulting in an increase of lipoteichoic acid (LTA) (19). LTA, a Gram-positive bacterial membrane component, can promote HCC development by enhancing senescent hepatic stellate cells (HSCs) (19). In addition, LTA function together with deoxycholic acid (DCA), a secondary bile acid produced by gut microbiota, to upregulate the expression of SASP factors and cyclooxygenase-2 (COX2) through Toll-like receptor 2 (TLR2), resulting in COX2-mediated prostaglandin E2 (PGE2) production to inhibit antitumor immunity (19, 20). Furthermore, obesity can modulate intestinal hormone secretion to impact liver function. For example, a high level of glucose-dependent insulinotropic polypeptide (GIP), an intestinal enteroendocrine K cell-secreted hormone, contributes to hepatic steatosis and liver injury by modulating the expression of microRNAs (miRNAs) (21). Overall, obesity can promote HCC progression through modulating metabolites, inflammation, immunity, and autophagy in the tumor microenvironment, as discussed below.

### Metabolites

Obesity-mediated metabolic change in the tumor microenvironment can suppress anti-tumor immunity (22). In addition, obesity induces the alteration of gut microbiota, which impacts anti-HCC immune therapies (23). For example, obesity induced by a HFD compromised the effect of cytotoxic CD8^+^ T cells in the tumor microenvironment by reprogramming fat intake in tumor cells *via* reducing prolyl hydroxylase-3 (PHD3) expression (7). Obesity can modulate glucose metabolism to promote HCC progression. Saturated fatty acids such as palmitate impact cancer stem cell properties, production of reactive oxidative species, and glucose metabolism to enhance HCC initiation and progression (24, 25). In addition, feeding a HFD promoted the production of lactate when the mice received glucose (25). T-regulatory cells (Tregs) can proliferate in lactate-rich environments, which mediates suppression effector T cell function (26). In addition, feeding a HFD in carcinogen diethylnitrosamine (DEN)-injected promoted the development of HCC compared to mice fed a control diet, with increased secretion of lactate (25).

### Chronic Inflammation

Low-grade chronic inflammation displays a key role in obesity and metabolic disorder (27). Inflammation impacts the activation of innate and adaptive immunity and modulates the progression of fibrosis and angiogenesis. For example, obesity-induced aberrant biosynthesis of glycosaminoglycan (GAG) which functions as one of the damage-associated molecular patterns (DAMPs) to promote hepatic inflammation and HCC *via* nuclear factor kappa B (NF-κB) signaling, in tumor suppressor gene exostosin-like 2-deficient mice (28). Adipose tissue caused by obesity can secrete diverse adipokines, such as leptin, adiponectin, and resistin, as well as proinflammatory cytokines, resulting in insulin resistance and chronic low-grade inflammation in different tissues, including liver tissue (29). For example, emerging evidence shows that leptin plays essential roles in cancer development by increasing tumor cell proliferation, metastasis, chemoresistance and promoting angiogenesis *via* binding its receptor to regulate many downstream signaling pathways (30, 31), as well as promoting NASH and liver fibrosis (32). Obesity impacts adipocytes to secrete proinflammatory cytokines such as interleukin (IL)-6 and tumor necrosis factor-α (TNF-α), and lipotoxicity induces hepatocyte death to activate Kupffer cells to produce those cytokines (33). Obesity-associated HCC development depended on the increased tumor-promoting cytokines IL-6 and TNF, inducing liver inflammation and activation of oncogenic signal transducer and activator of transcription 3 (STAT3) signaling in mice (34). The mRNA expression of Toll-like receptor 4 (TLR4) was positively correlated with IL-6 and IL-10 mRNA expression in obese HCC patients. Treatment with resatorvid, a TLR4 inhibitor, inhibited HCC growth in mice with deletion of phosphatase and tensin homolog (PTEN) in hepatocytes (35).

Plasma S100 calcium-binding protein A4 (S100A4) levels were positively associated with insulin resistance in prepubertal non-diabetic obese children, which has been shown to be associated with inflammation (36). Plasma levels of FGF-21 are higher in obese adolescents than lean controls, especially in those with fatty liver (37). Feeding a high fat, high sucrose (HFHS) diet, fibroblast growth factor 21 (FGF21) deficient mice developed advanced steatosis and liver fibrosis, with liver inflammation compared to wild-type mice (38).

### Immune Modulation

Obesity modulates intrahepatic immunity to induce an immunotolerant microenvironment, which results in HCC progression. For example, obese mice had a higher frequency of PD-1^+^ T cells in the liver compared to control mice (39). In addition, the expression of Ki67 in hepatic T cells in obese mice was reduced compared to that in control mice, post-*ex vivo* stimulation with anti-mouse CD3 antibody, as well as reduced interferon (IFN)-γ and TNF-α production, indicating functional exhaustion. The frequency of PD-1^+^ T cells and Ki67^+^ T cells in the peripheral blood of obese (BMI ≥ 30) volunteers were increased and decreased, respectively, compared to that in non-obese (BMI < 30) human healthy controls following *ex vivo* stimulation (39). Similarly, a recent study showed that PD1^+^CD8^+^ T cells were increased in the livers of mice with NASH, promoting NASH-HCC progression (40). In addition, Ma et al. reported that obesity caused hepatic lipid accumulation and loss of CD4^+^ T cells, which plays a critical role in NAFLD-HCC progression (41). A recent study from this group also showed that loss of liver CD4^+^ T cells impaired immunotherapies such as RNA vaccine (M30) and anti-OX40 antibody-mediated treatment against tumor cell growth in the liver (42). Furthermore, there are several other subtypes of T cells play important roles in the NALFD or NAFLD-HCC pathogenesis (43).

### Autophagy

Autophagy-related protein 4b (Apg4b)-deficient mice, with limited autophagy function, showed a bodyweight gain compared to wild-type mice when the mice were challenged with a high-fat diet consisting of 42% fat or with a standard rodent diet with 30% sucrose supplementation in drinking water (44). Apg4b-deficient mice also displayed more accumulation of visceral and hepatic fat, low glucose tolerance, and reduced insulin responses. By modulating autophagy, lipid metabolism, endoplasmic reticulum (ER) stress, and mitochondrial dysfunction can be regulated to ameliorate obesity-associated pathogenesis (45).

Excessive production of reactive oxygen species (ROS) plays a pivotal role in the pathogenesis of obesity, which promotes obesity-related metabolic disorders, including diabetes, NAFLD, and HCC (45). Autophagy can be turned off to rescue ROS-induced cell damage (46). Furthermore, autophagy-related genes were also shown to be aberrantly expressed in cholangiocarcinoma (47), the secondary most liver cancer. Treatment with hydroxychloroquine can induce cell apoptosis and inhibit cholangiocarcinoma cell proliferation by increasing ROS accumulation through inhibiting autophagy (47).

## T2DM and HCC

T2DM has been reported to be an independent factor that is associated with increased risk for HCC for both men and women in the U.S (48)., as well as Asian countries. For example, in Japan, the concurrence of HCC with T2DM and obesity is reported in hepatitis B virus surface antigen-negative or hepatitis C virus antibody-negative patients (49). Another report also showed that HCC-caused mortality was higher than other cancer-associated death in T2DM patients in Japan (50). Some differentially methylated genes (DMGs) were co-expressed in HCC and T2DM, such as ST3 beta-galactoside alpha-2,3-sialyltransferase 2 (ST3GAL2) and glycerophosphodiester phosphodiesterase domain containing 2 (GDPD2). And also, these DMGs were implicated in the signaling pathways including biosynthesis of glycosaminoglycan and unsaturated fatty acids (51). In addition, patients with NAFLD showed a higher risk of incident T2DM than those without NAFLD, even more in those with advanced high NAFLD fibrosis scores (52). Even after transarterial chemoembolization (TACE), a shorter interval time of progression and higher risk of cancer-specific mortality were found in HCC patients with T2DM who underwent TACE than patients without T2DM, especially in patients with cirrhosis (53).

A recent study showed that the prevalence of NAFLD in T2DM patients was 100%, while the prevalence of NASH was 96.82% (54). This study also showed that the HOMA-IR score (homeostatic model assessment for insulin resistance) was significantly higher in NASH patients than that in NALFD patients, which may cause a higher co-incidence of NASH with T2DM. Factors in T2DM pathogenesis impact HCC initiation and progression, including insulin/insulin-like growth factor (IGF) related factors (55, 56), proinflammatory cytokines (57), oxidative stress (58), gut microbiota dysbiosis (8, 59), angiogenesis (60), cell apoptosis (61), autophagy (62), which are summarized in **Table 1**.

**Table 1:** The underlying molecular mechanisms of HCC progression in T2DM patients or CVDs.

Factors	Function		References
**Type 2 diabetes mellitus (T2DM)**				
Insulin/insulin-like growth factor (IGF) related factors	Liver specific-knockout insulin receptor substrate (IRS) 1, one of the molecules responsible for insulin/IGF signaling transduce in the liver, reduced DEN-induced hepatocarcinogenesis and inflammation.		(55)
	HCC tumor cells-acquired resistance to sorafenib was associated with higher levels of IGF and fibroblast growth factor (FGF). Inhibiting IGF and FGF signaling pathways delayed tumor growth.		(56)
Proinflammatory cytokines	T2DM can induce liver inflammation evidenced by an increase of proinflammatory cytokines NF-κB, TNF-α, IL-6, and IL-1β, which is associated with cell apoptosis and oxidative stress, all factor promoting HCC progression.		(57)
Oxidative stress	Treatment with *Codonopsis lanceolata* polysaccharide improved high fat/high sucrose diet-induced insulin resistance *via* activating antioxidant nuclear factor erythroid 2-related factor 2 signaling and enzymes, such as superoxide dismutase and catalase.		(58)
Gut microbiota dysbiosis	Gut microbiota plays an important role in the pathogenesis of T2DM. For example, *Bifidobacterium* genus is commonly reported to be negatively associated with T2DM, while it was also reduced in NAFLD-related HCC patients.		(8, 59)
Angiogenesis	Treatment with sodium-glucose cotransporter 2 inhibitor (SGLT2i) canagliflozin (100 mg/day) induced a spontaneous regression of HCC in a cirrhotic patient with T2DM, with a reduction in angiogenesis-related cytokines, such as angiopoietin-1/2 and platelet-derived growth factor-AA (PDGF-AA).		(60)
Cell apoptosis	Hepatic expression of pro-apoptotic protein Bad was increased during the development of T2DM in mice, while anti-apoptotic protein Bcl-2 was not increased.		(61)
Autophagy	Treatment with fenofibrate, a peroxisome proliferator-activated receptor alpha (PPARα) agonist, can activate autophagy and reduce liver fat accumulation by upregulating transcription factors E3 and EB in HFD-fed mice.		(62)
**Cardiovascular diseases (CVDs)**			
Inflammation		Low-grade chronic inflammation, dysbiosis of gut microbiota, infection, and genetic factors can lead to the development of obesity and CVDs, as well as insulin resistance and NAFLD, factors causing HCC.	(63–65)
Oxidative stress		Oxidative stress closely associated with inflammation is another major contributor to CVDs due to lack of antioxidant enzymes, such as superoxide dismutase and glutathione peroxidase, and overexpression of reactive oxygen species-producing enzymes, such as NADPH oxidase.	(66, 67)
Gut microbiota and relative metabolites		High levels of gut-derived metabolite trimethylamine-N-Oxide (TMAO) increased the risk of cardiovascular disease, which is also associated with NASH and primary liver cancer.	(68–70)
Metabolic disorders		Increased plasma cholesterol, especially low-density lipoprotein cholesterol (LDL-C), is associated with a higher risk of coronary artery disease (CAD). Insulin resistance impacts systemic lipid metabolism, which can lead to high levels of plasma triglycerides and low levels of high-density of lipoprotein, associated with CVD development.	(71–73)
Cell apoptosis		Apoptotic factors such as Bax and Bcl-2 are shown to be altered in different CVDs, associated with the change of inflammation and oxidative stress. In addition to apoptosis, ferroptosis and pyroptosis with a robust inflammatory response also play important roles in the progression of CVDs.	(74–76)
Viral Infections		A study showed that viral infection (HCV and/or HBV) significantly increased the 10-year cardiovascular risk and CVD events in patients with metabolic-associated fatty liver disease (MAFLD).	(77)
Autophagy		Intracellular and extracellular signals triggered by autophagy are involved in the pathogenesis of CVDs, which can be regulated by epigenetic factors such as microRNAs and long non-coding RNAs.	(78)

## CVDS and HCC

Cardiovascular diseases (CVDs) are a group of disorders of the heart and blood vessels. CVDs and their outcomes include myocardial infarction, angina, transient ischemic attack, stroke, claudication, and heart failure (79). Chronic liver disease can increase the development of CVD events. For example, a meta-analysis of studies on the effects of NAFLD and the risk of CVD showed that patients with NAFLD have a significantly increased risk of fatal and non-fatal CVD incidences than those without NAFLD (80). Hepatokines such as α2-HS-glycoprotein secreted in the liver during NAFLD contribute to both CVDs and T2DM as distinct pathogenic factors from skeletal muscle and adipose tissue (81). Not only liver diseases can impact the progression of CVDs, but cardiovascular complications can in turn affect hepatic function and disease progression (66). There are many co-factors of CVDs and HCC, such as inflammation (63–65), oxidative stress (66, 67), gut microbiota and their relative metabolites (68–70), metabolic dysfunction (71–73), cell apoptosis (74–76), viral infections (77), and autophagy (78) (**Table 1**). Due to their correlation, medicines for the treatment of heart disease could be applied to treat liver disease. For example, statins, β-Hydroxy β-methylglutaryl-CoA (HMG-CoA) reductase inhibitors with function to reduce the risk of CVD morbidity and mortality, show a beneficial effect on liver disease, including NASH and HCC (82, 83).

## Signaling Pathways and Processes

Obesity, T2DM, and CVDs share some signaling pathways to promote NAFLD-related HCC progression. Thus, some examples of canonical signaling pathways and new findings will be discussed in the following context.

### Wnt/β-Catenin

Wnt/β-catenin signaling is implicated in adipose tissue lipogenesis (84), activation of hepatic stellate cells (HSCs) or liver fibrosis (85), and ischemic myocardium (86). In primary liver cancers, including HCC, Wnt/β-catenin signaling is often activated to induce cancer cell growth and metastasis (87).

### IKK-β/NF-κB

In obesity-associated HCC, liver inflammation and ER stress are associated with higher expression of inositol-requiring enzyme 1α (IRE1α). IRE1α, the unfolded protein response (UPR) signal transducer in ER, can activate nuclear factor kappa B kinase subunit beta (IKK-β)/NF-κB signaling pathway to promote TNF and IL-6 expression, resulting in HCC progression (88).

### MiRNAs

The expression of miR-34a was upregulated in fatty liver and palmitate acid (PA)-treated BNL CL.2 cells, which can induce hepatocyte senescence *via* downregulating cyclin-dependent kinase 6 (CDK6) expression (89). Hydrodynamic injection of miR-15a/16-1 (containing the miR-15a and miR-16-1) can prevent HCC in both protein kinase B (AKT)/Ras and c-Myc mice with overexpression of activated forms of AKT and NRas oncogenes (AKT/Ras) or c-Myc, *via* suppressing Tregs function to increase the effect of cytotoxic T cells (90). As the most abundant miRNAs in the liver, miR-122 has been reported to be significantly suppressed in HCC cell lines and tumor tissues. Overexpression of miR-122 can increase HCC cell radiosensitivity and sensitivity to chemotherapy medicines (91).

### FABP5/HIF-1α

Proteomics analysis showed that hypoxia-inducible factor-1 alpha (HIF-1α), a transcription factor, is a binding protein of fatty acid-binding protein 5 (FABP5) (92). In addition, fatty acid (e.g., oleic acid) can activate FABP5/HIF-1α signaling to modulate lipid metabolism reprogramming to promote HCC progression, as well as the proliferation of HCC cells.

### STAT3 Signaling Pathway

Signal transducer and activator of transcription 3 (STAT3) signaling pathway is involved in apoptosis, migration, and epithelial-mesenchymal transition (EMT) of HCC cells (93, 94). Surgical procedure-induced overexpression of IL-11 promoted tumor cell growth and recurrence of HCC *via* activating STAT3 signaling, while blocking IL-11/STAT3 signaling dampened HCC recurrence after surgical resection (95).

### PI3K/Akt

Aberrant activation and inhibition of phosphatidylinositol-4,5-bisphosphate 3-kinase (PI3K)/AKT signaling pathway are associated with HCC cell proliferation (96) and apoptosis (97), respectively. One study showed that protein arginine methyltransferase 9 can regulate EMT to increase HCC cell migration and invasion by activating Snail expression through PI3K/AKT signaling pathway (98).

### PPARs

Peroxisome proliferator-activated receptors (PPARs), including PPAR-α (99), PPAR-β/δ (100), and PPAR-γ (101), are involved in HCC growth and metastasis. PPARs can be regulated by microRNAs (miRNAs) to regulate tumor cell proliferation, migration, and invasion. For example, miR-1468 can promote HCC progression by activating PPAR-γ/AKT signaling pathway (102).

### VEGF/VEGFR Signaling

Vascular endothelial growth factor (VEGF) or its receptor (VEGFR) plays an important role in angiogenesis in HCC. A cohort study in Turkish showed that HCC patients with low levels of serum VEGF-A (<100 pg/mL) had a higher overall survival (OS) rate compared to patients with high levels of serum VEGF-A (≥100 pg/mL), indicating as an independent predictor for OS in HCC patients (103). Treatment of bioactive compound VS 8 can induce human HCC cell line HepG2 cell apoptosis and inhibit the expression of EMT-induced transcription factors in CD44^+^CD133^+^ cancer stem cells, by inhibiting VEGF/VEGFR-2 signaling pathway (104).

## Treatments

**Figure 1:**
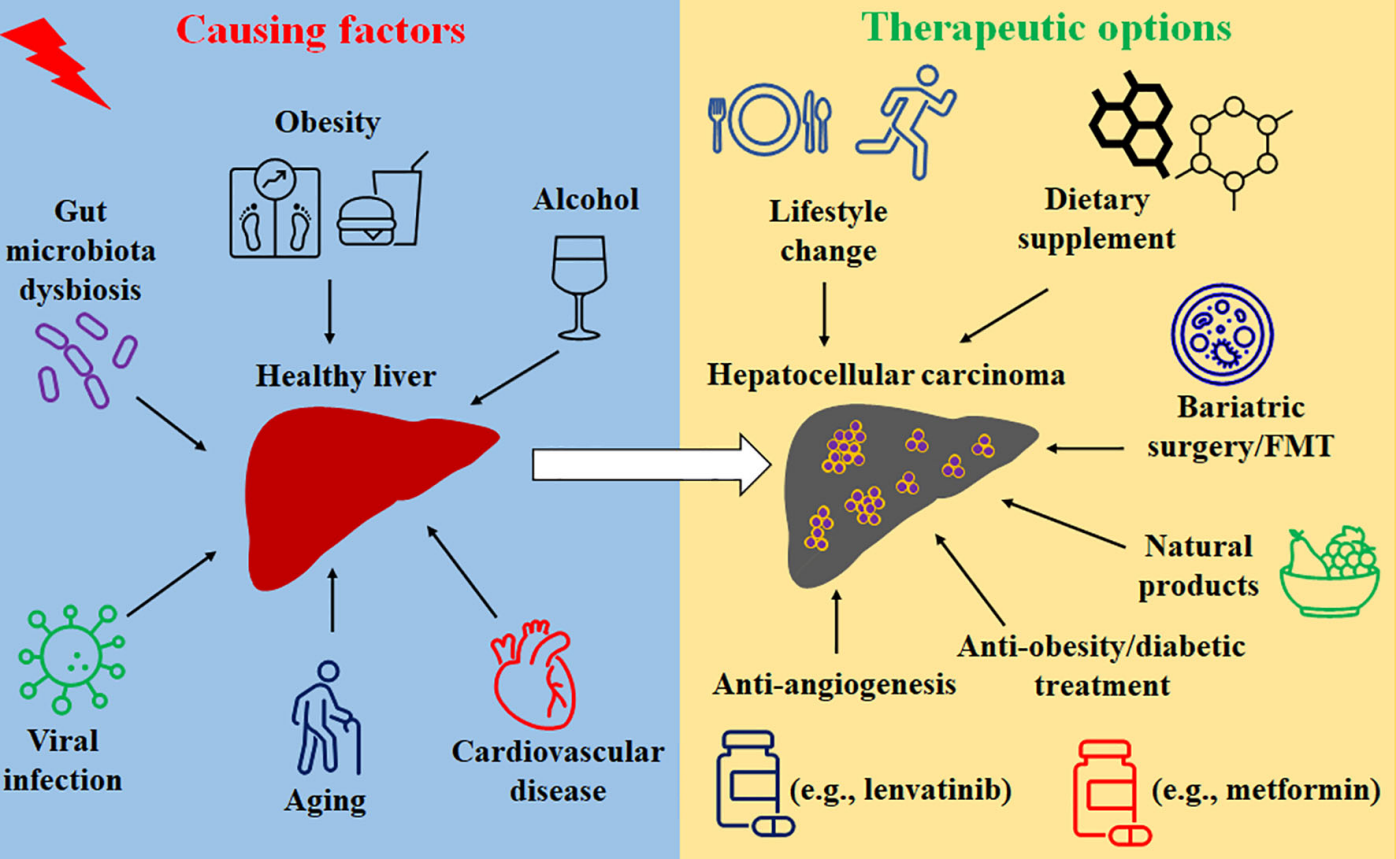
The causing factors and treatment options of HCC.

Prevention and treatment options for HCC include lifestyle change, dietary supplement, modulation of gut microbiota, anti-inflammation and anti-oxidative stress medicines, anti-obesity and anti-diabetic treatments, anti-angiogenesis, as well as natural products-mediated therapies, which are summarized in **Figure 1**.

### Lifestyle Change

Unhealthy lifestyles, such as over-nutrition, smoking, drinking, and lack of exercise, are risk factors causing cancer development and progression (105). One study showed that consumption of caffeinated coffee with an extra two cups daily was positively associated with reduction in the risk of HCC, which was also shown with consumption of decaffeinated coffee to some extent (106). Another study showed that intermediate-salt (6-10 g/day) or high-salt (>10 g/day) intake displayed a higher risk to develop primary liver cancer compared to low-salt intake (<6 g/day), after adjusting other potential cofactors such as fatty liver, hypertension, and diabetes (107). Lifestyle intervention can effectively result in low body fat mass, reduction of visceral adiposity, and a decrease of metabolic diseases, including NAFLD, CVD, and T2DM (108, 109); therefore, change of lifestyle plays an essential role in preventing HCC development.

Environmental or dietary exposure to aflatoxin B1, a genotoxic hepatocarcinogen, can also drive a high risk of HCC (110, 111). Aflatoxin contamination in food has been reported in food products, such as groundnuts, maize, wheat, and cocoa, which is associated with fungal growth (112). Therefore, consumption with fresh and non-contaminated food is also critically important to reduce potential risk of HCC.

### Dietary Supplement

Supplement of eicosapentaenoic acid (EPA), an omega-3 polyunsaturated fatty acid, reduced the development of obesity-related HCC in mice *via* suppressing the expression of STAT3 to inhibit tumor growth (113). Another study also omega-3 supplementation can decrease hepatic *de novo* lipogenesis while increasing fatty acid oxidation (114). Both *in vitro* and *in vivo* studies show that Se and selenoproteins exert immunomodulatory function against HCC by modulating oxidative stress, inflammation, angiogenesis, cell proliferation, and apoptosis (115).

### Bariatric Surgery and FMT

Strategies *via* modulating gut microbiota are able to change anti-cancer immune response and inhibit factors causing HCC development, including bariatric surgery (BS) and fecal microbiota transplantation (FMT). For example, BS can inhibit the onset of NASH and HCC in a large propensity-matched cohort study after 7.1 years of follow-up (116), which can ameliorate NASH features including steatosis, hepatocyte ballooning, and lobular inflammation (117). Another meta-analysis with a comprehensive literature review also showed that BS was associated with a decreased HCC risk (118). Another review paper explored the potential of FMT in the prevention of NAFLD/NASH and improving the anti-cancer immune response (119).

### Anti-Inflammation and Anti-Oxidative Stress

We and other researchers show that natural anti-oxidative and anti-inflammatory product astaxanthin can modulate intrahepatic and systemic inflammation and oxidative stress to inhibit NASH and liver fibrosis (120). Molecular mechanism study showed that metformin treatment inhibited the expression of IL-12-mediated proliferation, migration, and invasion of HCC cells and attenuated ectopic IL-22 expression-caused HCC progression by activating the Hippo signaling pathway (121). In addition, it has been shown that astaxanthin inhibits the alcoholic fatty liver disease (AFLD) *via* modulating gut microbiota, resulting in a decrease of phyla Bacteroidetes and Proteobacteria and genera *Butyricimonas*, *Bilophila*, and *Parabacteroides*, while inducing an increase of phylum Verrucomicrobia and genus *Akkermansia* compared to control group (122).

Aspirin, a nonsteroidal anti-inflammatory drug, can reduce pain, fever, and reduce the risk of a heart attack. A prospective study showed that daily aspirin use inhibited the progression of NASH and advanced fibrosis in NAFLD patients (123). In addition, Ricciotti et al. reported that as an adjuvant, aspirin has the ability to reduce the recurrence of HCC, which is associated with anti-inflammatory and antiplatelet functions (124).

### Anti-Obesity and Anti-Diabetic Treatments

A retrospective study showed that the incidence of HCC was significantly lower in T2DM patients with cirrhosis with metformin treatment (17.4% in total of 125 patients) compared to patients without metformin treatment (37.4% in total of 128 patients) (125). For HCC patients, metformin treatment extended the median survival time from 3.88 years to 6.9 years (125). Another meta-analysis study showed that metformin treatment can significantly prolong the OS of HCC patients with T2DM after curative therapy (126). Metformin treatment can reduce HCC risk, but the effective dose has racial disparity in HCC patients with T2DM but without chronic liver disease (127).

Several studies showed that the combination of anti-diabetic drug liraglutide with human umbilical cord mesenchymal stem cell (hUC-MSCs) can modulate glycolipid metabolism, insulin resistance, and liver injury in rats with T2DM *via* inhibiting pancreatic beta-cell apoptosis, TLR4/NF-κB signaling pathway, and oxidative stress (128, 129). Furthermore, cholesterol-lowering drugs such as statin show beneficial effects against HCC development (130) or recurrence (131).

### Anti-Angiogenesis

Angiogenesis resulting from an imbalance of factors such as VEGF/VEGFR signaling can advance HCC progression. The approved anti-angiogenic drugs (AAD) such as sorafenib, regorafenib, and lenvatinib have been shown to have a therapeutic effect on HCC (5). However, proteinuria caused by AAD can impact their effect against HCC. Angiotensin-converting enzyme inhibitors have been applied to reduce AAD-related proteinuria (132), but these inhibitors show inhibiting effects to the efficacy of AADs. In addition, some studies showed that T2DM patients with HCC who received metformin are resistant to sorafenib treatment (133, 134), having poor progression-free survival (PFS) and OS.

### Natural Products-Mediated Therapies

Treatment with hirsutine, an indole alkaloid isolated from *Uncaria rhynchophylla*, can attenuate HFD-induced hepatic steatosis, peripheral hyperglycemia, cardiac hypertrophy, and insulin resistance, *via* activating PI3K/AKT pathway (135). Another study showed that freshly dried mulberry fruits can avoid hyperphagia and reduce body weight gain and visceral fat accumulation, ameliorating hypertrophy of arterial and cardiac walls, aortic collagen fiber, and hepatic lipid accumulation in HFD-fed mice (136).

Furthermore, eradication of viral infection is also helpful to reduce metabolic disorders. For example, hepatitis C virus (HCV) eradication treated with direct-acting antivirals can reduce the incidence of T2DM by improving insulin resistance and restoring glucose homeostasis altered during viral infection (137). In addition, HCV clearance was also independently associated with a decreased risk of cardiovascular events (138), as well as major cardiovascular events in prediabetic patients (139). Therefore, anti-HCV treatment is helpful for metabolic disease-associated progression of HCC.

## Conclusions

NAFLD comorbidities obesity, T2DM, and CVDs are risk factors that contribute to HCC initiation and progression. The incidence of metabolic disease-associated HCC is increased in the past decade, due to the increase of NAFLD and its comorbidities. Pathogenic factors such as abnormal metabolites, inflammatory factors, and immune modulations are underlying mechanisms for metabolic dysfunction associated with HCC pathogenesis. Currently, many treatment options show promising effects in HCC. However, the benefit of treatments such as sorafenib is still limited. In addition, an inappropriate combination of treatments even may reduce the effect of monotherapy. More clinical trials are awaited to explore the potential treatments for metabolic disease-associated HCC. A better understanding of the underlying mechanism of how these metabolic dysfunctions promote HCC initiation and progression is helpful to provide precision medicine care personally.

## Author Contributions

Conceptualization and data collection: CZ, SL, and MY. Original draft preparation, review, and editing: CZ, SL, and MY. All authors contributed to the article and approved the submitted version.

## Conflict of Interest

The authors declare that the research was conducted in the absence of any commercial or financial relationships that could be construed as a potential conflict of interest.

## Publisher’s Note

All claims expressed in this article are solely those of the authors and do not necessarily represent those of their affiliated organizations, or those of the publisher, the editors and the reviewers. Any product that may be evaluated in this article, or claim that may be made by its manufacturer, is not guaranteed or endorsed by the publisher.
